# Peripheral Arterial Stiffness Increases the Risk of Progression of Renal Disease in Type 2 Diabetic Patients

**DOI:** 10.3389/fmed.2020.588967

**Published:** 2020-09-30

**Authors:** Tae Hoon Lim, Seung Min Chung, Dong Sung Lee, Se Ra Choi, Jun Sung Moon, Ji Sung Yoon, Kyu Chang Won, Hyoung Woo Lee

**Affiliations:** ^1^College of Medicine, Yeungnam University, Daegu, South Korea; ^2^Division of Endocrinology and Metabolism, Department of Internal Medicine, Yeungnam University Medical Center, Daegu, South Korea

**Keywords:** arterial stiffness, diabetes mellitus, diabetic nephropathy, pulse wave velocity, renal function decline

## Abstract

**Aims:** Our aim was to investigate the effects of peripheral arterial stiffness on the risk of progression of renal disease in patients with type 2 diabetes (T2D).

**Methods:** This was a single center, retrospective cohort study. Brachial-ankle pulse wave velocity (baPWV) tests were performed on T2D patients in 2015. Increased arterial stiffness was defined as baPWV of ≥ 1800 cm/s. We applied criteria for progression of renal disease according to EMPA-REG OUTCOME trial.

**Results:** In total, 186 patients were enrolled in the final study. The mean age was 59.1 years and male:female ratio was 1.73:1. Thirteen (7%) patients progressed to renal disease during the average follow-up time of 35.3 months. In particular, the risk of progression to macroalbuminuria was significantly higher in the baPWV ≥ 1800 cm/s group (HR 6.216, *p* = 0.020). Individuals with a baPWV of ≥ 1800 cm/s (when comparisons were adjusted for age, sex, blood pressure, diabetes duration, eGFR, and use of renin-angiotensin system inhibitors) had a significantly higher risk of the progression of renal disease (HR = 8.480, *p* = 0.014).

**Conclusion:** These results suggest that peripheral arterial stiffness (baPWV ≥ 1800 cm/s) may be a risk factor for the progression of renal disease in T2D patients.

## Introduction

Type 2 diabetes mellitus (T2D), along with hypertension, is one of the leading chronic diseases in Korea (1). In addition to the increasing prevalence of diabetes, diabetic complications such as cardiovascular disease, retinopathy, neuropathy, and nephropathy have significant negative effects on quality of life among T2D patients.

Aside from the microvascular complications that affect T2D patients, diabetic kidney disease (DKD) is the most common cause of end-stage renal disease (ESRD) resulting in the increased morbidity and mortality of diabetic patients. According to the Korean Society of Nephrology, for the past 20 years diabetes has been identified as the leading cause of ESRD requiring renal replacement treatment, accounting for 48.8% of cases (2). ESRD causes a heavy socioeconomic and health burden (3), so it is important to closely monitor T2D patients and try to preserve renal function, as the progression of renal disease is often irreversible (4). Recent guidelines strongly recommend the annual screening of renal function, which can be determined by measuring urinary albumin, blood creatinine, and glomerular filtration rate from the time of diagnosis (4, 5). Previous clinical trials have shown that close monitoring and controlling of glucose levels and blood pressure can aid in delaying the progression of DKD, but some clinical needs remain a challenge.

Arterial stiffness is often a consequence of the physiological aging process or atherosclerosis, and it has been also described as a biomarker of hypertension (6), subclinical (7) or overt cardiovascular disease, stroke (8), and mortality (9) in the general population. Arterial stiffness is best characterized by measuring the pulse wave velocity (PWV), which is a commonly used biomarker (10–13). Previous studies have found that PWV can predict CKD progression and mortality (14, 15). Additionally, the level of PWV is significantly higher in people with diabetes compared to those without (16, 17). However, few studies have explored the possible causal relationship between PWV and the progression of diabetic kidney disease in patients with T2D.

To address this research gap, we investigated the relationship between arterial stiffness and renal dysfunction in T2D patients, as well as whether brachial-ankle pulse wave velocity (baPWV) can be used clinically as a predictor of the progression of renal disease.

## Subjects, Materials and Methods

### Study Population

This was a retrospective cohort study, in which data were collected from the electronic medical records of T2D patients who underwent baPWV testing in 2015 and were subsequently assessed for the progression of renal disease from 2016–2019 at Yeungnam University Hospital. In total, 391 patients initially qualified for this study. Patients were excluded for any of the following reasons: not aged 21–79 years (*n* = 7); missing initial estimated glomerular filtration rate data (eGFR; *n* = 51); chronic kidney disease stage <3b (eGFR ≤ 45 ml/min/1.73 m^2^); treated with renal replacement therapy at baseline (*n* = 9); presence of peripheral arterial stenosis confirmed by an ankle brachial index (ABI) of <0.9 (*n* = 5) (18, 19); or follow-up of renal function was not possible (*n* = 133). In total, 186 patients were finally determined to be eligible. The study protocol was developed in accordance with the tenets of the Declaration of Helsinki and reviewed and approved by the institutional review board of the Yeungnam University Hospital (IRB no. 2020-05-020).

### Anthropometrics and Laboratory Measurements

Body mass index (BMI) was calculated as weight divided by height squared (kg/m^2^). Data on diabetes duration, comorbidities (hypertension, stroke, and coronary artery disease), and use of renin-angiotensin system (RAS) inhibitors were collected. All laboratory tests were performed in the central laboratory of the Yeungnam University Hospital. Venous blood sampling was taken from the antecubital vein after an overnight fast. The levels of serum glucose, glycated hemoglobin (HbA1c), total protein, albumin, aspartate aminotransferase (AST), alanine aminotransferase (ALT), total cholesterol, triglyceride, high-density lipoprotein (HDL) cholesterol, low-density lipoprotein (LDL) cholesterol, blood urea nitrogen (BUN), and creatinine were measured. The levels of urine creatinine and albumin were also measured.

### Measurement and Definition of Arterial Stiffness

The blood pressure (BP), baPWV, and ABI were measured using a non-invasive vascular screening device (BP-203RPEIII, OMRON healthcare, Japan), which was operated by a trained examiner who placed pneumatic cuffs on each ankle and each upper arm of patients in the supine position. The validity of baPWV compared to aortic PWV using invasive catheter manometer was reported to be favorable (*r* = 0.87, *p* < 0.01) (20). We defined a person with a baPWV of ≥ 1800 cm/s as having a higher level of arterial stiffness, in accordance with the Japanese Circulation Society. A baPWV of ≥ 1800 cm/s is regarded as the threshold for a high-risk category (18) and is known to increase the risk for cardiovascular and heart failure-related events (7, 21).

### Measurement and Definition of the Progression of Renal Disease

Estimated glomerular filtration rate (eGFR) was calculated using the chronic kidney disease epidemiology collaboration equation (CKD-EPI). Urinary albumin:creatinine ratio (uACR) was calculated. In accordance with the EMPA-REG OUTCOME trial, any of the following criteria were used to describe progression of renal disease (22): progression to macroalbuminuria (uACR > 300 mg); doubling of serum creatinine level accompanied by an eGFR of ≤ 45 ml/min/1.73 m^2^; treatment with renal replacement therapy; or death from renal disease.

### Statistical Analysis

Numerical data are expressed as the mean ± standard deviation (SD), and categorical data are expressed as numbers and percentages. The independent *t*-test was used to compare the groups with continuous variables. The chi-square test was used to compare categorical variables. A Cox proportional hazards regression was used to evaluate the risk of progression of renal disease. IBM Statistical Package for Social Sciences for Windows, version 21.0 (SPSS Inc. Chicago, IL) software was used for statistical analyses. Graphs were produced using GraphPad Prism 8.0 software (GraphPad Software Inc., San Diego, CA, USA). A value of *p* < 0.05 was considered statistically significant.

## Results

### Comparison of Baseline Characteristics Between High and Low Pulse Wave Velocity

The mean age of patients was 59.1 ± 10.5 years (range 22–79) and the male:female ratio was 1.73:1. The mean duration of diabetes was 9 ± 7.8 years (range 0–39). The prevalence of comorbid hypertension, stroke, and coronary artery disease in the patient cohort was 57.5, 6.5, and 4.3%, respectively. 23.7% of enrolled patients (*n* = 44) had a baPWV of ≥ 1800 cm/s.

**Figure 1:**
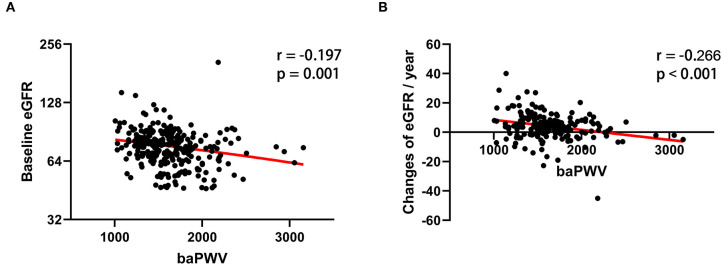
Scatter plot of the relationship between baPWV and **(A)** baseline eGFR and **(B)** annual changes in eGFR. The red line represents the regression line.

Table 1 lists baseline characteristics according to baPWV (≥ 1800 cm/s vs. <1800 cm/s). Individuals with a baPWV of ≥ 1800 cm/s were significantly older (*p* < 0.001), had longer duration of diabetes (*p* = 0.002), higher systolic and diastolic blood pressure (*p* < 0.01), and higher ABI (*p* = 0.02) compared to patients with a baPWV of <1800 cm/s. Factors including sex, BMI, comorbid hypertension, stroke or coronary artery disease, and use of RAS inhibitor medication did not differ significantly between the two groups. Baseline HbA1c, albumin, liver and lipid profiles also did not differ significantly between the two groups. In the baPWV of ≥ 1800 cm/s group, eGFR was significantly lower (72.6 vs. 80.8 ml/min/1.73 m^2^, *p* = 0.005) and uACR was higher but without statistical significance (92.2 vs. 55.7 mg/g, *p* = 0.172). Table 2 lists correlations between baPWV and age, diabetes duration, systolic blood pressure, and eGFR at baseline. Age, diabetes duration, and systolic blood pressure were positively correlated with baPWV (*r* = 0.405, 0.213, and 0.645, respectively; all *p* < 0.01), whereas baPWV was negatively correlated with eGFR (*r* = −0.193, *p* = 0.011; Figure 1A).

**Table 1:** Baseline characteristics according to the presence of peripheral arterial stiffness (baPWV ≥ 1800 cm/s).

	**baPWV <1800**	**baPWV ≥1800**	***p* value**
	**cm/s (*n* = 142)**	**cm/s (*n* = 44)**	
Age (yrs)	57.4 ± 10.2	64.3 ± 9.5	**<0.001**
Male, *n* (%)	95 (66.9%)	23 (52.3%)	0.078
Diabetes duration (yrs)	8.1 ± 7.3	12.2 ± 8.5	**0.002**
BMI (kg/m^2^)	24.8 ± 5.0	24.1 ± 3.2	0.258
Hypertension *n* (%)	76 (71.0%)	31 (70.5%)	0.091
Stroke *n* (%)	10 (7.0%)	2 (4.5%)	0.556
Coronary artery disease *n* (%)	5 (3.5%)	3 (7.0%)	0.329
RAS inhibitors, *n* (%)	65 (45.8%)	27 (61.4%)	0.071
Systolic BP (mmHg)	125.8 ± 15.4	145.3 ± 19.1	**<0.001**
Diastolic BP (mmHg)	77.0 ± 10.2	83.7 ± 10.8	**<0.001**
baPWV (cm/s)	1490.1 ± 194.0	2118.6 ± 308.2	**<0.001**
ABI	1.15 ± 0.08	1.17 ± 0.09	**0.020**
HbA1c (%)	8.3 ± 2.3	8.6 ± 1.7	0.230
Total protein (g/dl)	7.3 ± 0.7	7.4 ± 0.5	0.781
Albumin (g/dl)	4.7 ± 0.6	4.7 ± 0.4	0.835
AST (unit/L)	28.7 ± 17.5	28.0 ± 10.1	0.899
ALT (unit/L)	31.6 ± 21.9	28.2 ± 15.4	0.300
Total cholesterol (mg/dl)	186.1 ± 59.9	188.1 ± 50.7	0.634
Triglyceride (mg/dl)	194.4 ± 181.9	175.2 ± 110.6	0.520
HDL cholesterol (mg/dl)	51.1 ± 15.5	50.6 ± 13.3	0.822
LDL cholesterol (mg/dl)	98.6 ± 48.5	101.3 ± 42.4	0.510
Creatinine (mg/dl)	1.01 ± 0.20	1.02 ± 0.22	0.603
**eGFR** (ml/min/1.73 m^2^)	80.8 ± 16.4	72.6 ± 14.3	**0.005**
uACR (mg/g)	55.7 ± 163.1	92.2 ± 172.6	0.172

*BMI, body mass index; RAS, renin-angiotensin system; BP, blood pressure; baPWV, brachial-ankle pulse wave velocity; ABI, ankle brachial index; AST, aspartate aminotransferase; ALT, alanine aminotransferase; HDL, high-density lipoprotein; LDL, low-density lipoprotein; eGFR, estimated glomerular filtration rate; uACR, urinary albumin:creatinine ratio. The bold values mean statistically significant data*.

**Table 2:** Correlation between baPWV and multiple variables at baseline.

	**baPWV**	
	***r***	***P*-value**
Age	0.405	<0.001
Diabetes duration	0.213	0.004
Systolic blood pressure	0.645	<0.001
eGFR	−0.193	0.011

### Comparison of the Risk for the Progression of Renal Disease Between High and Low Pulse Wave Velocity

**Figure 2:**
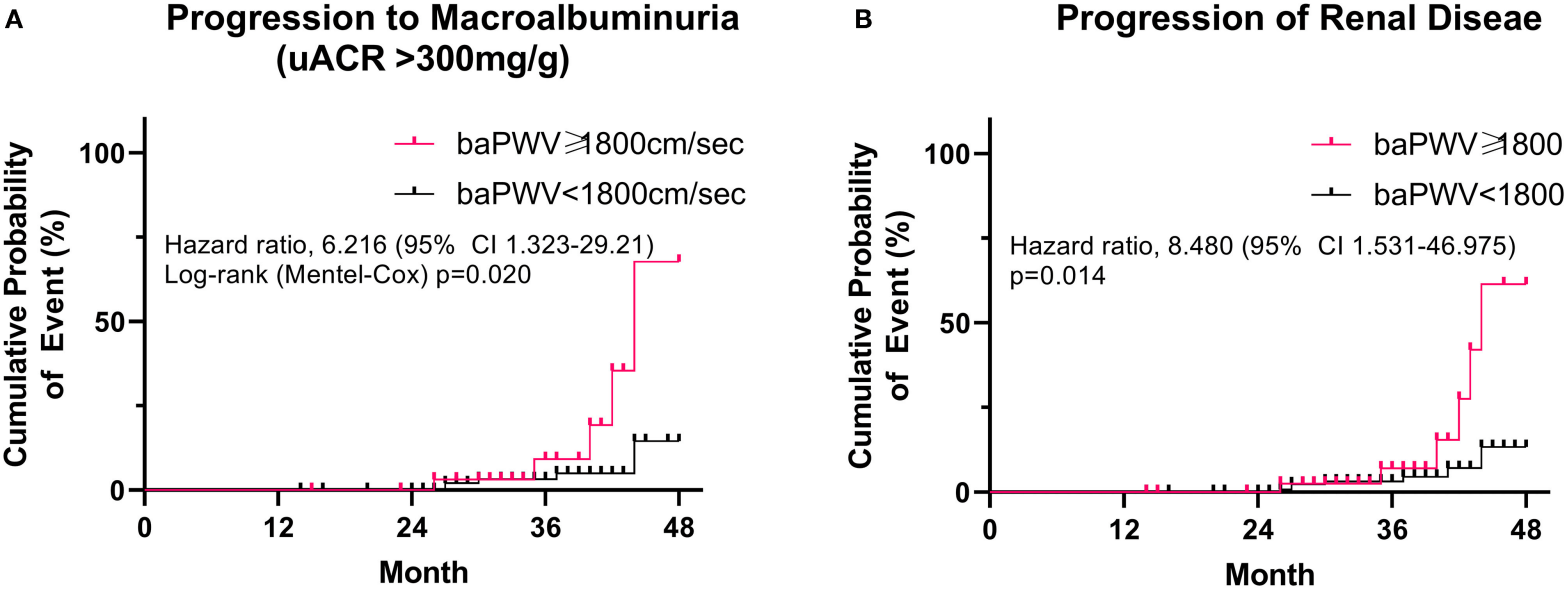
Risk of peripheral arterial stiffness (baPWV ≥ 1800 cm/s) on **(A)** progression to macroalbuminuria (uACR > 300 mg) and **(B)** progression of renal disease. A log-rank (Mantel-Cox) survival analysis was used.

The mean follow-up period was 35.3 ± 7.25 months (range 14–48). During the follow-up period, 13 patients developed renal disease (13.6% of the baPWV of ≥ 1800 cm/s group and 4.9% of the baPWV of <1800 cm/s group). Specifically, 10 patients progressed to macroalbuminuria and 3 patients exhibited doubling of serum creatinine levels accompanied by an eGFR of ≤ 45 ml/min/1.73 m^2^. No patients were treated with renal replacement therapy or died from renal disease. The annual changes in eGFR were significantly negatively correlated with baPWV (*r* = −0.226, *p* < 0.001; Figure 1B), meaning that the higher the baPWV, the greater the annual decrement of eGFR. Individuals with a baPWV of ≥ 1800 cm/s had a significantly higher risk for progression to macroalbuminuria (HR 6.216, 95% CI 1.323–29.21, *p* = 0.020; Figure 2A), whereas no significant risk was observed for the doubling of serum creatinine level accompanied by an eGFR of ≤ 45 ml/min/1.73 m^2^ (HR 2.762, 95% CI 0.146–52.07, *p* = 0.497). The additional analysis of comparing the progressors and non-progressors regarding macroalbuminuria group are presented in Table 3. Compared to the non-progressor group, baseline baPWV was significantly higher in the progressor group (1627.8 vs. 1831.2 cm/s, *p* = 0.041). And although not statistically significant, the duration of diabetes was longer (8.8 vs. 12.8 years, *p* = 0.051) and the rate of taking RAS inhibitors were higher (47.7 vs. 80.0%, *p* = 0.056) in the progressor group. Table 4 lists the risk of arterial stiffness for progression of renal disease based on a Cox-regression analysis. Age, sex, systolic BP, diastolic BP, diabetes duration, eGFR, use of RAS inhibitors, and baPWV were considered as covariates. In non-adjusted regression analysis, the lower baseline eGFR, and baPWV of ≥ 1800 cm/s increased the risk of progression of renal disease. After adjustments, individuals with a baPWV of ≥ 1800 cm/s had an increased risk for progression of renal disease compared to those with a baPWV of <1800 cm/s (HR 8.480, 95% CI 1.531-46.975, *p* = 0.014; Figure 2B).

**Table 3:** Characteristics according to progressors and non-progressors regarding albuminuria.

	**Non-progressors**	**Progressors**	***p* value**
	**(*n* = 176)**	**(*n* = 10)**	
Age (yrs)	58.9 ± 10.5	61.5 ± 10.6	0.401
Male, *n* (%)	110 (62.5%)	8 (80.0%)	0.330
Diabetes duration (yrs)	8.8 ± 7.9	12.8 ± 5.5	0.051
RAS inhibitors, *n* (%)	84 (47.7%)	8 (80.0%)	0.056
Systolic BP (mmHg)	130.4 ± 18.6	131.2 ± 11.4	0.623
Diastolic BP (mmHg)	78.7 ± 10.8	75.1 ± 9.1	0.425
baPWV (cm/s)	1627.8 ± 348.5	1831.2 ± 334.8	**0.041**
ABI	1.16 ± 0.08	1.17 ± 0.11	0.913
HbA1c (%)	8.3 ± 2.1	9.0 ± 2.2	0.199
eGFR (ml/min/1.73m^2^)	75.9 ± 18.3	77.7 ± 12.4	0.465

*Mann-Whitney U test, Fisher's exact test was used. The bold values mean statistically significant data*.

**Table 4:** Risk of peripheral arterial stiffness (baPWV ≥ 1800 cm/s) on the progression of renal disease.

	**Non-adjusted model**			Adjusted model^*^		
	**HR**	**95% CI**	***p*-value**	**HR**	**95% CI**	***p*-value**
Age	1.008	[0.954-1.064]	1.008	0.965	[0.898-1.037]	0.330
Male	3.078	[0.681-13.913]	0.144	5.009	[0.838-29.952]	0.077
Systolic BP	0.986	[0.953-1.021]	0.443	0.993	[0.923-1.068]	0.847
Diastolic BP	0.962	[0.909-1.019]	0.180	0.926	[0.823-1.041]	0.197
Diabetes duration	1.038	[0.983-1.095]	0.185	1.017	[0.945-1.094]	0.653
Baseline eGFR	0.977	[0.959-0.995]	**0.015**	1.003	[0.986-1.020]	0.741
RAS inhibitor medication	2.598	[0.789-8.559]	0.116	2.097	[0.576-7.631]	0.261
baPWV ≥1800 cm/s	3.271	[1.096-9.769]	**0.034**	8.480	[1.531-46.975]	**0.014**

Any one of the following criteria was used to describe the progression of renal disease: progression to macroalbuminuria, doubling of serum creatinine levels accompanied by an estimated glomerular filtration rate (eGFR) of ≤ 45 ml/min/1.73 m^2^, treatment with renal replacement therapy, or death from renal disease.

* *Adjusted for age, sex, systolic BP, diastolic BP, diabetes duration, baseline eGFR, use of RAS inhibitors, and baPWV. The bold values mean statistically significant data*.

## Discussion

After following patients for an average duration of 35 months, we found that T2D patients with a baPWV ≥ 1800 cm/s are at 8.5-fold greater risk of progression of renal disease, regardless of classical risk factors. This finding suggests that peripheral arterial stiffness represented by a high baPWV may accelerate the progression of CKD in patients with type 2 diabetes.

Many previous studies have suggested that arterial (or aortic) stiffness is closely related with the risk of mortality and specifically cardiovascular disease, not only in the general population but also in patients with underlying comorbidities such as hypertension and diabetes (6, 8–10). The CRIC study (Chronic Renal Insufficiency Cohort) revealed that high aortic stiffness increases the risk of chronic kidney disease progression and all-cause death in patients with impaired kidney function (23). Other studies have demonstrated that arterial stiffness is increased in patients with T2D (24) and that PWV can be used as a predictor for cardiovascular events and all-cause mortality (17). Arterial stiffness may cause left ventricular hypertrophy and dysfunction due to higher pressure pulse pulsation (19). A plausible explanation for the impact of arterial stiffness on the progression of renal disease is that arterial stiffness increases hemodynamic shear stress, which may result in endothelial dysfunction and microvascular ischemia, ultimately causing kidney injury (25, 26).

High PWV has been shown to be associated with diabetic retinopathy (27), neuropathy (28, 29), and nephropathy. A Rotterdam study of 3,666 subjects from the general population included an 11-year follow-up and revealed that carotid-femoral pulse wave velocity (cfPWV) was associated with a rapid reduction in eGFR and CKD progression (14). Another 9-year follow-up of 211 T2D patients in the UK revealed that cfPWV was associated with a decrease in eGFR among patients under 60 years of age (30). A study in Singapore involving 1012 T2D patients of differing Asian ethnicities and an average follow-up period of 3.1 years revealed that cfPWV was an independent predictor for albuminuria progression (31). These results indicate that cfPWV can be a predictor of DKD progression in T2D. Nevertheless, a study involving 913 subjects from the general Korean population and an average follow-up period of 3.2 years found that neither cfPWV nor baPWV are related to renal dysfunction and that the significance of baPWV in T2D patients is likely invalid due to the relatively small proportion of T2D patients (32). The most important aspect of the present study is that baPWV was associated with a decrease in renal function when Korean patients with T2D were followed-up for an average of 3 years. Additionally, the present study used the validated cut-off value for baPWV and demonstrated that this association exists, despite applying very strict criteria for the progression of renal disease.

A study of 461 Japanese T2D patients, with an average of 5.9 years follow-up, investigated the risks of arterial stiffness on progression of renal disease in T2D. It found that patients with a cfPWV of ≥ 910 cm/s were at increased risk of albuminuria (transition from normo- to micro-albuminuria or from micro- to macro-albuminuria; HR = 1.23), and a linear regression analysis revealed a negative correlation between cfPWV and annual change in eGFR (33). Our study produced similar results: patients with a baPWV of ≥ 1800 cm/s were at increased risk of progression of renal disease; specifically, progression to macroalbuminuria and the annual change of eGFR were negatively correlated with baPWV. In addition to cfPWV, baPWV would be a useful screening tool for predicting progression of renal disease in T2D patients, due to the ease and convenience of measurements.

This study had a limitation that it enrolled a relatively small number of patients and used the single measurement of baPWV. However, to our knowledge, this is the first study to identify the risk of peripheral arterial stiffness on the progression of renal disease with the capability of distinguishing a causal relationship.

In conclusion, baPWV representing peripheral arterial stiffness can be used as a predictor of the progression of renal disease in T2D patients.

## Data Availability Statement

The raw data supporting the conclusions of this article will be made available by the authors, without undue reservation.

## Ethics Statement

The studies involving human participants were reviewed and approved by Institutional review board of the Yeungnam University Hospital. The patients/participants provided their written informed consent to participate in this study.

## Author Contributions

SMC and JSM: conceptualization and study design. THL, DSL, and SRC: data collection. THL, SMC, DSL, and SRC: data analysis and interpretation. THL, SMC, and JSM: writing. JSY, KCW, and HWL: review and editing. All authors contributed to the article and approved the submitted version.

## Conflict of Interest

The authors declare that the research was conducted in the absence of any commercial or financial relationships that could be construed as a potential conflict of interest.

## References

[1] Won JC Lee JH Kim JH Kang ES Won KC Kim DJ. Diabetes fact sheet in Korea, 2016: an appraisal of current status. Diabetes Metab J. (2018) 42:415–24. 10.4093/dmj.2018.0017 30113146 PMC6202557

[2] ESRD Registry Committee: Korean Society of Nephrology Current Renal Replacement Therapy in Korea. (2019). Available online at: http://www.ksn.or.kr/rang_board/list.html?code=sinchart_eng

[3] Kim SH Jo MW Go DS Ryu DR Park J. Economic burden of chronic kidney disease in Korea using national sample cohort. J Nephrol. (2017) 30:787–93. 10.1007/s40620-017-0380-3 28303461

[4] National Kidney F. KDOQI clinical practice guideline for diabetes and CKD: 2012 update. Am J Kidney Dis. (2012) 60:850–86. 10.1053/j.ajkd.2012.07.005 23067652

[5] American Diabetes Association. Microvascular Complications and Foot Care: Standards of Medical Care in Diabetes-2020. Diabetes Care. (2020). 43(Suppl 1):S135–51. 10.2337/dc20-S011 31862754

[6] Oh YS Arterial stiffness and hypertension. Clin Hypertens. (2018) 24:17 10.1186/s40885-018-0102-8 30519485 PMC6271566

[7] Chung YK Lee YJ Kim KW Cho RK Chung SM Moon JS. Serum cystatin C is associated with subclinical atherosclerosis in patients with type 2 diabetes: A retrospective study. Diab Vasc Dis Res. (2018) 15:24–30. 10.1177/1479164117738156 29090609

[8] Mattace-Raso FU van der Cammen TJ Hofman A van Popele NM Bos ML Schalekamp MA. Arterial stiffness and risk of coronary heart disease and stroke: the Rotterdam Study. Circulation. (2006) 113:657–63. 10.1161/CIRCULATIONAHA.105.555235 16461838

[9] Cruickshank K Riste L Anderson SG Wright JS Dunn G Gosling RG. Aortic pulse-wave velocity and its relationship to mortality in diabetes and glucose intolerance: an integrated index of vascular function? Circulation. (2002) 106:2085–90. 10.1161/01.CIR.0000033824.02722.F7 12379578

[10] Yamashina A Tomiyama H Arai T Hirose K Koji Y Hirayama Y. Brachial-ankle pulse wave velocity as a marker of atherosclerotic vascular damage and cardiovascular risk. Hypertens Res. (2003) 26:615–22. 10.1291/hypres.26.615 14567500

[11] Covic A Haydar AA Bhamra-Ariza P Gusbeth-Tatomir P Goldsmith DJ. Aortic pulse wave velocity and arterial wave reflections predict the extent and severity of coronary artery disease in chronic kidney disease patients. J Nephrol. (2005) 18:388–96. 16245242

[12] Munakata M Sakuraba J Tayama J Furuta T Yusa A Nunokawa T. Higher brachial-ankle pulse wave velocity is associated with more advanced carotid atherosclerosis in end-stage renal disease. Hypertens Res. (2005) 28:9–14. 10.1291/hypres.28.9 15969249

[13] Avolio A. Arterial stiffness. Pulse (Basel). (2013) 1:14–28. 10.1159/000348620 26587425 PMC4315342

[14] Sedaghat S Mattace-Raso FU Hoorn EJ Uitterlinden AG Hofman A Ikram MA. Arterial Stiffness And Decline In Kidney Function. Clin J Am Soc Nephrol. (2015) 10:2190–7. 10.2215/CJN.03000315 26563380 PMC4670762

[15] van Varik BJ Vossen LM Rennenberg RJ Stoffers HE Kessels AG de Leeuw PW. Arterial stiffness and decline of renal function in a primary care population. Hypertens Res. (2017) 40:73–8. 10.1038/hr.2016.113 27604344

[16] Taniwaki H Kawagishi T Emoto M Shoji T Kanda H Maekawa K. Correlation between the intima-media thickness of the carotid artery and aortic pulse-wave velocity in patients with type 2 diabetes. Vessel wall properties in type 2 diabetes. Diabetes Care. (1999) 22:1851–7. 10.2337/diacare.22.11.1851 10546019

[17] Cardoso CR Ferreira MT Leite NC Salles GF. Prognostic impact of aortic stiffness in high-risk type 2 diabetic patients: the Rio deJaneiro Type 2 Diabetes Cohort Study. Diabetes Care. (2013) 36:3772–8. 10.2337/dc13-0506 23877987 PMC3816863

[18] Munakata M. Brachial-ankle pulse wave velocity in the measurement of arterial stiffness: recent evidence and clinical applications. Curr Hypertens Rev. (2014) 10:49–57. 10.2174/157340211001141111160957 25392144

[19] Kim HL Kim SH Pulse wave velocity in atherosclerosis. Front Cardiovasc Med. (2019) 6:41 10.3389/fcvm.2019.00041 31024934 PMC6465321

[20] Yamashina A Tomiyama H Takeda K Tsuda H Arai T Hirose K. Validity, reproducibility, and clinical significance of noninvasive brachial-ankle pulse wave velocity measurement. Hypertens Res. (2002) 25:359–64. 10.1291/hypres.25.359 12135313

[21] Takae M Yamamoto E Tokitsu T Oike F Nishihara T Fujisue K. Clinical significance of brachial-ankle pulse wave velocity in patients with heart failure with reduced left ventricular ejection fraction. Am J Hypertens. (2019) 32:657–67. 10.1093/ajh/hpz048 31090886

[22] Wanner C Inzucchi SE Lachin JM Fitchett D von Eynatten M Mattheus M Empagliflozin and progression of kidney disease in type 2 diabetes. N Engl J Med. (2016) 375:323–34. 10.1056/NEJMoa1515920 27299675

[23] Townsend RR Anderson AH Chirinos JA Feldman HI Grunwald JE Nessel L. Association of pulse wave velocity with chronic kidney disease progression and mortality: findings from the CRIC Study (Chronic Renal Insufficiency Cohort). Hypertension. (2018) 71:1101–7. 10.1161/HYPERTENSIONAHA.117.10648 29712736 PMC6342478

[24] Schram MT Henry RM van Dijk RA Kostense PJ Dekker JM Nijpels G. Increased central artery stiffness in impaired glucose metabolism and type 2 diabetes: the Hoorn Study. Hypertension. (2004) 43:176–81. 10.1161/01.HYP.0000111829.46090.92 14698999

[25] Chung SM Oh JH Moon JS Kim YK Yoon JS Won KC Critical shear stress is associated with diabetic kidney disease in patients with type 2 diabetes. Sci Rep. (2018) 8:908 10.1038/s41598-018-25252-8 29343776 PMC5772353

[26] Safar ME London GM Plante GE. Arterial stiffness and kidney function. Hypertension. (2004) 43:163–8. 10.1161/01.HYP.0000114571.75762.b0 14732732

[27] Yun YW Shin MH Lee YH Rhee JA Choi JS. Arterial stiffness is associated with diabetic retinopathy in Korean type 2 diabetic patients. J Prev Med Public Health. (2011) 44:260–6. 10.3961/jpmph.2011.44.6.260 22143176 PMC3249265

[28] Ha BK Kim BG Kim DH Lee SI Jung SM Park JY. Relationships between brachial-ankle pulse wave velocity and peripheral neuropathy in type 2 diabetes. Diabetes Metab J. (2012) 36:443–51. 10.4093/dmj.2012.36.6.443 23275938 PMC3530715

[29] Wu N Cai X Ye K Li Y He M Zhao W. Association between Brachial-Ankle pulse wave velocity and cardiac autonomic neuropathy in type 2 diabetes. Diabetol Metab Syndr. (2014) 6:82. 10.1186/1758-5996-6-82 25126115 PMC4132900

[30] Fountoulakis N Thakrar C Patel K Viberti G Gnudi L Karalliedde J. Increased arterial stiffness is an independent predictor of renal function decline in patients with type 2 diabetes mellitus younger than 60 years. J Am Heart Assoc. (2017) 6:4. 10.1161/JAHA.116.004934 28360227 PMC5533009

[31] Zhang X Low S Sum CF Tavintharan S Yeoh LY Liu J. Arterial stiffness is an independent predictor for albuminuria progression among Asians with type 2 diabetes-A prospective cohort study. J Diabetes Complications. (2017) 31:933–8. 10.1016/j.jdiacomp.2017.02.004 28392041

[32] Kim CS Kim HY Kang YU Choi JS Bae EH Ma SK. Association of pulse wave velocity and pulse pressure with decline in kidney function. J Clin Hypertens (Greenwich). (2014) 16:372–7. 10.1111/jch.12302 24716575 PMC8031886

[33] Bouchi R Babazono T Mugishima M Yoshida N Nyumura I Toya K. Arterial stiffness is associated with incident albuminuria and decreased glomerular filtration rate in type 2 diabetic patients. Diabetes Care. (2011) 34:2570–5. 10.2337/dc11-1020 21972413 PMC3220850

